# Generation of tumor spheroids using a droplet-based microfluidic device for photothermal therapy

**DOI:** 10.1038/s41378-020-0167-x

**Published:** 2020-06-29

**Authors:** Jong Min Lee, Ji Wook Choi, Christian D. Ahrberg, Hyung Woo Choi, Jang Ho Ha, Seok Gyu Mun, Sung Joon Mo, Bong Geun Chung

**Affiliations:** 1 Department of Mechanical Engineering, Sogang University, Seoul, Korea; 2 Division of Chemical Industry, Yeungnam University College, Daegu, Republic of Korea; 3 Research Center, Sogang University, Seoul, Korea; 4 Department of Biomedical Engineering, Sogang University, Seoul, Korea

**Keywords:** Nanoparticles, Microfluidics

## Abstract

Despite their simplicity, monolayer cell cultures are not able to accurately predict drug behavior in vivo due to their inability to accurately mimic cell-cell and cell-matrix interactions. In contrast, cell spheroids are able to reproduce these interactions and thus would be a viable tool for testing drug behavior. However, the generation of homogenous and reproducible cell spheroids on a large scale is a labor intensive and slow process compared to monolayer cell cultures. Here, we present a droplet-based microfluidic device for the automated, large-scale generation of homogenous cell spheroids in a uniform manner. Using the microfluidic system, the size of the spheroids can be tuned to between 100 and 130 μm with generation frequencies of 70 Hz. We demonstrated the photothermal therapy (PTT) application of brain tumor spheroids generated by the microfluidic device using a reduced graphene oxide-branched polyethyleneimine-polyethylene glycol (rGO-BPEI-PEG) nanocomposite as the PTT agent. Furthermore, we generated uniformly sized neural stem cell (NSC)-derived neurospheres in the droplet-based microfluidic device. We also confirmed that the neurites were regulated by neurotoxins. Therefore, this droplet-based microfluidic device could be a powerful tool for photothermal therapy and drug screening applications.

## Mass production of miniature medical models

A microfluidic device for the efficient production of three-dimensional ‘spheroid’ cell cultures could accelerate the discovery and testing of therapeutic agents. These small clumps of cells can better recapitulate the physiology of the tissue environment than two-dimensional cultures, but are challenging to produce in a high-throughput fashion. Researchers led by Prof. Bong Geun Chung of Sogang University, Seoul, Korea have now developed a system that can generate 42,000 spheroids/h with minimal user intervention. As an initial demonstration, they produce brain tumor-derived spheroids, and use these to test the cancer-killing efficacy of a therapy based on heated graphene-based nanoparticles. Prof. Chung and colleagues subsequently employ their device to assess the detrimental effects of the neurotoxin thapsigargin on neural stem cell-derived spheroids, highlighting the potential value of this platform for high-throughput cell screening assays.

## Introduction

Cell experiments are generally conducted using monolayer cell cultures. However, due to their inability to reproduce cell-cell and cell-matrix interactions as well as the absence of physico-biochemical barriers, drug efficiencies tend to be overestimated in monolayer cell cultures^1^. For example, the half maximal inhibitory concentration (IC_50_) of paclitaxel, a drug active against tumors, was found to be two orders of magnitude greater in a three-dimensional (3D) culture than in a two-dimensional (2D) monolayer culture^2^. Due to their better reproduction of cell-cell, cell-matrix, and physico-biochemical barriers, spheroids have gained increasing popularity in cell culture experiments. Conventionally, cell spheroids have been formed using a number of methods, such as pellet culture^3^, liquid overlay^4^, hanging drop^5^, spinning flask^6^, and magnetic levitation methods^7^. Despite their reliable formation of spheroids, these methods suffer from a wide distribution of spheroid diameters, laborious handling procedures, low-throughput, and static environments leading to the fast depletion of oxygen and nutrients^8^.

Through advances in microfabrication techniques, microfluidics can offer solutions to the issues typically encountered in 3D spheroid culture. Microfluidic approaches for the generation and culture of cell spheroids can be classified into three different categories: microwell, microtrap, and droplet-based approaches^9^. In microwell spheroid cultures, an array of microwells is created in a hydrogel^10^. Alternatively, micropatterns of hydrophilic spots on a hydrophobic surface can be used to generate large arrays of aqueous compartments for the spheroid culture^11^. Through this method, uniform-sized spheroids can be generated in a high-throughput fashion, requiring a comparatively small amount of manual handling. Moreover, the spheroid size can be controlled by varying the diameter of the microwells used. Enclosing the microwells in a microfluidic device allows for the facile exchange of growth medium during spheroid culture, preventing nutrient or oxygen depletion^12^. Similar devices can further be used to co-culture spheroids simply by encapsulating two cell lines within the microwells^13^. As an alternative to microwells, U-shaped cell traps can also be used for capturing cells and forming spheroids with a well-defined number of cells^14^. Despite their advantages and applications, U-shaped cell traps still suffer from a relatively low generation yield, expertise requirements, and automation limits. To overcome these limitations, the microfluidic-based droplet, as a third option, has been used for the generation of spheroids in a rapid and highly efficient manner. Apart from micropatterned systems, which require another chip fabrication to adjust the spheroid size, the microfluidic-based droplet allows for prominent control of spheroid size. Additionally, a large number of spheroids can be generated by encapsulating cells inside droplets, and droplets with homogeneous volumes can be created at a higher frequency in an automated manner. Alginate is added to the droplets to allow polymerization of the droplet-containing cells. The solidified droplets can then be removed from the oil phase and the spheroids can be cultured in growth medium^15^. The further introduction of magnetic beads into the droplets allows for facile separation of the solidified droplets after polymerization from the oil phase with a magnetic field^16^. Breaking up the cell clusters inside the microfluidic device prior to the formation of droplets allows for a more homogenous distribution of cells encapsulated within the droplets^17^. Utilizing the laminar flow inside the microfluidic devices, core-shell spheroids of two different cell types can be produced^18^. While spheroids have gained increasing popularity for cell experiments, nanomaterials have become a popular choice for cancer therapy due to the advantages provided by their nanometer size. For example, graphene oxide (GO) nanosheets can be used for photothermal therapy (PTT) due to their absorption of light in the near infrared (NIR) region of the spectrum and small size that allows their uptake by cancer cells^19,20^. Through grafting of polyethylene glycol (PEG) onto GO nanosheets, the stability of the nanosheets can be increased, and the tendency to aggregate decreases, leading to an increase in therapeutic efficiency^21^. Further chemical modification of GO nanosheets allows for the creation of nanomaterials combining different therapeutic approaches into a single material. For example, branched polyethyleneimine (BPEI) crafted onto GO nanosheets can be used for photothermal-triggered drug delivery in combined chemo-photothermal therapy^22^. A similar nanomaterial has also been used for gene delivery, combining photothermal therapy and gene therapy. To develop these novel nanomaterials and evaluate their therapeutic efficiency, a 3D cell spheroid is a valuable tool.

Here, we describe a droplet-based microfluidic device for the generation of 3D cell spheroids at high frequencies with minimal manual handling requirements. The spheroids are initially cultured inside droplets in a microfluidic device before harvesting. The harvested spheroids are used to evaluate the PTT effects of reduced GO (rGO)-BPEI-PEG nanocomposites to demonstrate the applications of the generated 3D cell spheroids for drug testing applications. As a proof-of-concept, we further tested drug screening to investigate the effect of neurotoxins on neurite outgrowth from uniform-sized neural stem cell (NSC)-derived neurospheres.

## Results and discussion

### Microfluidic device for generating tumor spheroid droplets

**Fig. 1:**
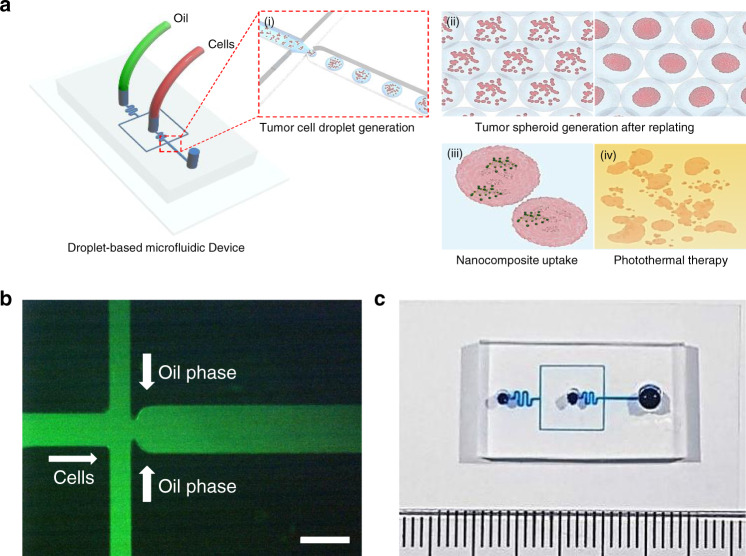
Generation of 3D tumor spheroids using the drop-based microfluidic device for photothermal therapy. **a** Schematic illustration of the generation of the 3D tumor spheroids and photothermal therapy. **b** Microscopic image of the droplet generation junction; for illustration purposes. the microfluidic channels were filled with a green dye. The scale bar is 300 μm. **c** Photograph of the fabricated droplet-based microfluidic device

**Fig. 2:**
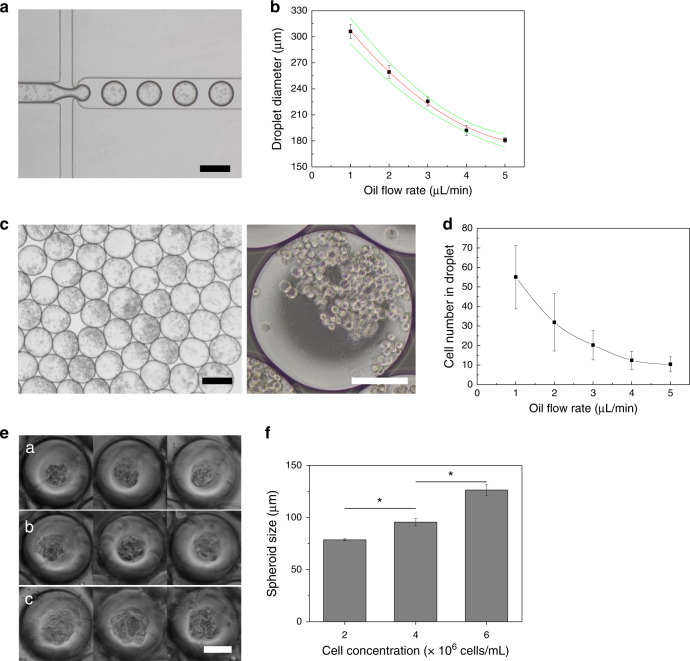
Generation and analysis of tumor spheroids. **a** Microscope image of the generation of tumor droplets. **b** Graph of droplet diameter against oil flow rate at a constant water flow rate of 1 μL/min. **c** Microscope image of the collected cell droplets. **d** Graph of cells encapsulated in the individual droplets against oil flow rate. **e** Microscopic images of the generated tumor spheroid after 24 h in the droplet. **f** Quantitative analysis of tumor spheroid size by cell concentration. All scale bars are 100 μm

The droplet-based microfluidic device consisted of two inlets for the oil phase and aqueous phase containing the cells (Fig. 1a, c). For droplet generation, a microfluidic junction was used to generate aqueous droplets containing U87MG glioblastoma cells through shear forces (Fig. 1b)^23,24^. To prevent contact of the aqueous phase containing the cells with the channel walls, flow rates were chosen so that the maximum diameter of the droplets was below the diameter of the channel (300 μm, Fig. 2a). By varying the flow rate of the fluorinated oil phase, the droplet diameter could be changed. The droplet diameters decreased as the oil flow rate increased (Fig. 2b). Thus, droplet diameters between 180.8 ± 2.9 and 305.9 ± 8.1 μm can be accessed by the microfluidic system. The generated droplets with the enclosed cells could then be used for spheroid culture (Fig. 2c). The droplets generated from the microfluidic device were transferred to well plates through the tube and subsequently cultured in an incubator for an additional 2 days (Supplementary Fig. S1a, b). After spheroid formation, the diameter (D) and shape index (*ShI*) of the spheroids were calculated as previously described^25^. These parameters classified tumor spheroids (*D* > 50 μm, ShI > 0.7), cell aggregates (*D* > 50 μm, ShI < 0.7), cell units (9 < *D* < 50 μm), and empty droplets (*D* < 9 μm) (Supplementary Fig. S1c). We confirmed that one spheroid per droplet constituted a large proportion of the population (~80%), and there were no droplets containing more than one spheroid per droplet (Supplementary Fig. S1d). To control the size of the spheroids, the number of cells must be controlled. For this, the microfluidic device offers two options. First, the number of cells in the droplet can be controlled by the droplet volume, which can be easily adjusted by the flow rate of the continuous phase (Fig. 2d). Alternatively, the concentration of cells in the dispersed phase can be adjusted (Fig. 2e, f). Using cell concentrations between 2×10^6^ and 6×10^6^ cells/mL, cell spheroid sizes ranging from 98.6 ± 1.0 to 126.4 ± 4.9 μm could be achieved. The droplet generation frequency of the microfluidic system was ~70 Hz under the fastest condition (oil flow rate of 50 μL/min), allowing for the generation of 42,000 droplets for spheroid culture within 10 min and showing ~20% higher generation yield than previously reported work^1^ (Supplementary Video 1). These data illustrate how the microfluidic system can be used to generate droplets for spheroid culture in a high-throughput manner. The diameter of the spheroids can be easily controlled by either changing the flow rate of the dispersed phase or by changing the concentration of the cells in the dispersed phase. These results demonstrate the significant advantages of our droplet-based microfluidic system compared to previous existing systems (Supplementary Table S1).

### Synthesis and characterization of the rGO-BPEI-PEG nanocomposites

**Fig. 3:**
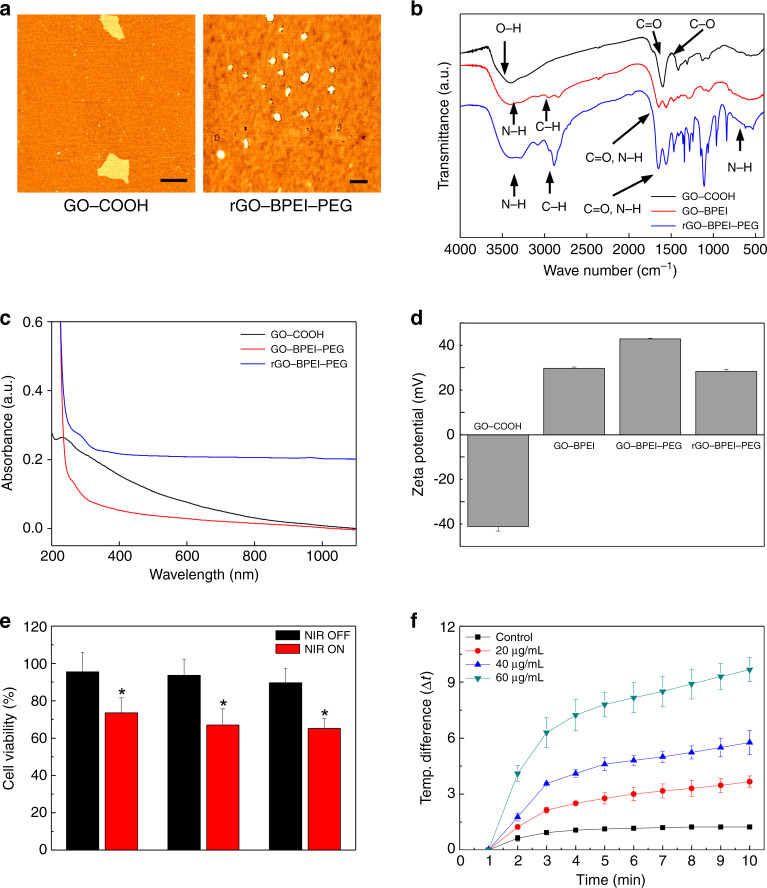
Characterization and photothermal effects of the rGO-BPEI-PEG nanocomposites. **a** AFM images of the GO-COOH and rGO-BPEI-PEG nanocomposites. Scale bars are 100nm. **b** FT-IR spectra of the GO-COOH, rGO-BPEI and rGO-BPEI-PEG nanocomposites. **c** UV-vis spectra of the GO-COOH, rGO-BPEI and rGO-BPEI-PEG nanocomposites. **d** Zeta potential measurements of the GO-COOH, GO-BPEI, rGO-BPEI and rGO-BPEI-PEG nanocomposites. **e** Cell viability of U87MG brain tumors after NIR laser treatment with rGO-BPEI-PEG nanocomposites and control experiments without the addition of rGO-BPEI-PEG nanocomposites. **f** Graph of the temperature increase after irradiating different solutions of rGO-BPEI-PEG nanocomposites with NIR laser irradiation

A nanocomposite was synthesized to demonstrate a possible application of the spheroids in this study. For this purpose, GO was reduced to increase its photothermal effect, and PEG was conjugated to increase colloidal stability, resulting in the synthesis of rGO-BPEI-PEG nanocomposites. The size and morphology of the rGO-BPEI-PEG nanocomposites were confirmed by atomic force microscopy (AFM) (Fig. 3a). The size of the unmodified GO-COOH was approximately 100–150 nm, and its shape was that of a nanosheet. However, rGO-BPEI-PEG was found to have a round shape, and its size was reduced to 50–60 nm. The decreased size of rGO-BPEI-PEG could be attributed to the folding and reforming of the GO nanosheet during the conjugation process of BPEI and PEG by the EDC/NHS reaction^26^. The conjugation between the carboxyl group of GO and the amino group of BPEI and the chemical reduction of rGO-BPEI-PEG were analyzed by FT-IR spectroscopy (Fig. 3b). GO-COOH showed a broad band at ~3361 cm^−1^ corresponding to O–H stretching vibrations. The stretching adsorption band originating from carboxyl groups (C = O) in the GO-COOH units was observed at 1728 cm^−1^, indicating the introduction of carboxyl groups onto the GO nanosheet. The chemical conjugation of BPEI on GO was confirmed by the new characteristic peaks of amide bonds at 1630-1695 cm^−1^, C–H vibration at 2900 cm^−1^, and N–H vibration at 670 and 3250 cm^−1^. After conjugation of PEG onto GO-BPEI, the –CH_2_ and –CH_3_ peaks were observed at 1466 and 1340 cm^−1^ in PEG, respectively. The FT-IR spectrum also showed –C–O–C– asymmetric and symmetric stretching at 1097 and 960 cm^−1^, respectively^27^. Moreover, the broad band at ~3360 cm^−1^ corresponded to O–H stretching vibrations, and this absorption was diminished in rGO-BPEI-PEG, indicating the successful chemical reduction of GO-BPEI-PEG. To demonstrate the potential of photothermal therapy using GO-based nanomaterials, we investigated the optical properties of GO-COOH, GO-BPEI-PEG, and rGO-BPEI-PEG by UV-visible spectroscopy (Fig. 3c). Reduction of GO-BPEI-PEG with hydrazine monohydrate induced higher UV-visible absorbance than that observed for the unreduced GO-COOH and GO-BPEI-PEG, which could be attributed to the restoration of the conjugated aromatic clusters^28,29^. The surface charges of GO-COOH, GO-BPEI, GO-BPEI-PEG, and rGO-BPEI-PEG were determined by using a zeta potential analyzer (Fig. 3d). Carboxylated GO showed a negative surface charge (−41 mV), and this value had a greater negative value than that of pure GO due to the presence of an increased number of carboxyl groups^30^. After conjugation of BPEI and PEG on the GO surface, the zeta potential values were observed to be positive (30 and 43 mV), which clearly demonstrated the chemical conjugation of BPEI and PEG onto the carboxylated surface^31^. rGO-BPEI-PEG showed a slightly decreased zeta potential value compared with that of GO-BPEI-PEG (+28 mV), indicating that the remaining hydroxyl and epoxy groups on GO-BPEI-PEG were completely removed by the chemical reduction process.

### PTT effects of rGO-BPEI-PEG nanocomposites on 3D cancer spheroids

**Fig. 4:**
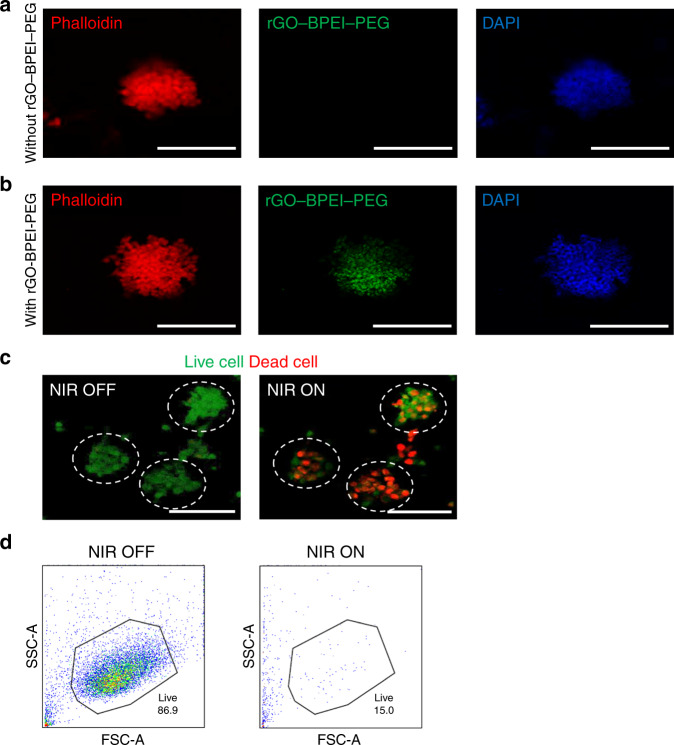
Analysis of PTT-treated brain tumor spheroids. **a** Fluorescence microscopy images of brain tumor spheroids a without rGO-BPEI-PEG. **b** Fluorescence microscopy images of brain tumor spheroids with rGO-BPEI-PEG nanocomposites. **c** Fluorescence microscopy images of brain tumor spheroids before and after NIR laser treatment. Live and dead cells were stained with calcein AM (green) and ethidium homodimer (red). **d** FACS cell viability analysis of control and brain tumor spheroids in PTT. All scale bars are 100 μm

To describe a clear biological definition of spheroids, we further performed immunostaining of cell adhesion molecules using E-cadherin, which is used as a cell-cell junction marker (Supplementary Fig. S2b). The immunostaining results showed high levels of E-cadherin expression in the tumor spheroids, indicating that spheroids were formed within the droplet. Due to the unique physical and chemical properties of graphene-based nanocomposites^32^, rGO-BPEI-PEG nanocomposites can be used as PTT agents. First, we evaluated the toxicity of rGO-BPEI-PEG nanocomposites using a CCK-8 assay. U87MG brain tumors were treated with different concentrations of rGO-BPEI-PEG nanocomposites (20, 40, 60 μg/mL) for 4 h and then irradiated with 808 nm NIR laser at a power density of 1 W/cm^2^. As shown in Fig. 3e, the cell viability was ~90% at 60 μg/mL rGO-BPEI-PEG, showing no cytotoxicity from the rGO-BPEI-PEG nanocomposites. In contrast, after NIR irradiation, cell viability was reduced to below 60% in cells treated with 60 μg/mL rGO-BPEI-PEG nanocomposites. Second, we investigated the cellular uptake of U87MG brain tumors using rGO-BPEI-PEG nanocomposites. For this, single tumor cells and tumor spheroids were replated from droplets in the microfluidic device and subsequently treated with fluorescein isothiocyanate (FITC)-labeled rGO-BPEI-PEG nanocomposites at a concentration of 60 μg/mL for 4 h. Confocal microscopy images showed a significant increase in green fluorescence in the brain tumors, indicating successful uptake of the rGO-BPEI-PEG nanocomposites (Supplementary Fig. S3 and Fig. 4a,b). Third, the PTT effects of rGO-BPEI-PEG nanocomposites on 3D brain tumor spheroids were evaluated. The replated 3D brain tumor spheroids were incubated in medium containing 60 μg/mL rGO-BPEI-PEG nanocomposite and were then irradiated with a NIR laser (Fig. 4c). Fluorescence-activated cell sorting (FACS) results demonstrated that the viability of the 3D brain tumor spheroids treated with rGO-BPEI-PEG nanocomposites was significantly reduced after NIR laser irradiation (Fig. 4d). The viability of the tumor spheroids in response to NIR laser irradiation was measured after rGO-BPEI-PEG nanocomposite treatment (Supplementary Fig. S4). The viability of the tumor spheroids treated with rGO-BPEI-PEG nanocomposites decreased from 91% to 55% after NIR laser irradiation. This result suggested that the rGO-BPEI-PEG nanocomposite could be a suitable PTT agent for 3D tumor spheroids obtained from droplets.

### Drug screening of uniform-sized neurospheres

**Fig. 5:**
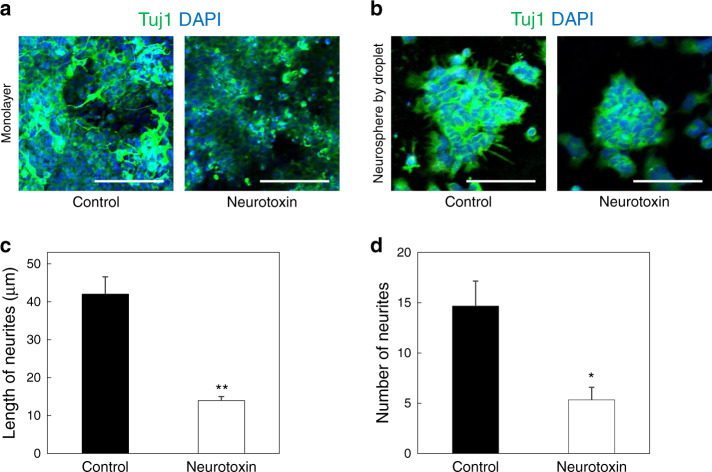
Drug screening analysis of neurospheres generated from droplet-based microfluidic devices. **a** Confocal laser scanning microscopy image of a neuronal cell monolayer. **b** Confocal laser scanning microscopy images of the neurosphere control and neurosphere treated with thapsigargin neurotoxin in the droplet-based microfluidic device. Scale bars are 100 μm. **c**, **d** Quantitative analysis of the length and numbers of neurites from the neurospheres

To study the effects of a neurotoxin on neurite outgrowth from uniform-sized neurospheres, we treated 1 μM thapsigargin neurotoxin with NSC-derived neurospheres generated from the droplet-based microfluidic device (Fig. 5). Thapsigargin-induced neurite damage was evaluated by immunostaining to investigate the effects of the neurotoxin on neuronal cells. The results confirmed that the Tuj1 intensity of the neuronal cell monolayer decreased after thapsigargin treatment (Fig. 5a). We also observed the thapsigargin-mediated neuronal damage on uniformly sized neurospheres (Fig. 5b). We found that the average lengths of neurites were reduced from 42 to 14 µm (Fig. 5c) and that the number of neurites significantly decreased after thapsigargin was treated with neurospheres (Fig. 5d). Therefore, this droplet-based microfluidic device can also be useful for various neurological disease drug screening applications.

## Conclusions

We developed a droplet-based microfluidic device to generate uniform-sized 3D brain tumor spheroids (100–130 μm in diameter) in a high-throughput manner. The frequency of droplet generation allows for 42,000 brain tumor spheroids/h, and the size of the tumor spheroids can be controlled without manual handling. We also demonstrated the PTT effect of rGO-BPEI-PEG nanocomposites on 3D brain tumor spheroids and the neurotoxin effect on uniform-sized neurospheres harvested from droplets from the microfluidic device. Therefore, our droplet-based microfluidic device has a number of advantages compared to conventional methods for the generation of tumor spheroids. First, spheroids can be generated in droplet-based microfluidic devices in a high-throughput manner, showing high yield (70 Hz). Second, our device can easily be automated, and the size of spheroids can be controlled with minimal manual handling. Third, our device can be used for the analysis of rGO-BPEI-PEG nanocomposite-mediated photothermal therapy of 3D tumor spheroids and drug screening of NSC-derived uniform-sized neurospheres.

## Materials and methods

### Fabrication of the droplet-based microfluidic device

The droplet-based microfluidic device was fabricated by a photolithography process, as previously described^33^. Briefly, the microfluidic device was designed by AutoCAD software. Silicon wafers (Wangxing Silicon-Peak Electronics, China) were spin-coated with SU-8 50 photoresist (Microchem Corp., USA) at 1000 rpm for 60 s, resulting in a thickness of 100 μm. Afterwards, the coated wafers were exposed to UV light for 30 s through a mask film. Poly(dimethylsiloxane) (PDMS, Dow Corning, USA) was mixed in a ratio of 10:1 (monomer:crosslinker), degassed, poured into the fabricated molds, and cured in an oven at 80 °C for 1 h. The cured PDMS devices were carefully separated from the silicon wafers and bonded onto glass substrates (Marienfeld, Germany) using oxygen plasma treatment (Femto Scientific, Korea). Before use, the channels of the PDMS device were treated with Aquapel (PPG Industries, USA) for 10 min to increase the hydrophobicity.

### Generation of the 3D tumor spheroids and neurospheres

Glioblastoma cells were cultured in Dulbecco’s modified Eagle’s medium (DMEM, Thermo Fisher Scientific, USA) with 10% fetal bovine serum (FBS, Thermo Fisher Scientific, MA, USA) and 1% penicillin-streptomycin (Thermo Fisher Scientific, USA) in a humidified incubator with 5% CO_2_ at 37 °C. For the generation of spheroids, the cells in growth medium were adjusted to the required cell concentration using growth medium and injected into the microfluidic device using a syringe pump. As a continuous phase, fluorinated oil (Novec7500, 3 M, Maplewood, USA) mixed with 2% diluted surfactant (Pico-Surf™, Dolomite Microfluidics, Royston, UK) was used, as previously shown^34^. To demonstrate droplet generation yield, we used deionized (DI) water with 10 v/v% blue food dye (Star Brand food color, FairPrice, Singapore). The generation yield was calculated by dividing the flow rates of the dispersed phase by the droplet volume. Generated droplets containing cells were collected at the outlet of the microfluidic device and incubated for an additional 2 days in the incubator for spheroid formation within the droplets. NE-4C NSCs were cultured in DMEM/F12 medium (Gibco, USA) containing 20 ng/mL basic fibroblast growth factor (bFGF) (R&D Systems, USA), 20 ng/mL epidermal growth factor (EGF) (Invitrogen, USA), 1% N-2 supplement, 2% B27 supplement, and 1% penicillin-streptomycin (Gibco, USA). NSC-induced neurospheres were formed by the same procedure as the 3D tumor spheroid and were subsequently replated onto poly-l-ornithine (PLO) and laminin-coated glass after 2 days. To induce neuronal differentiation, the differentiation medium without EGF and bFGF was changed every 2 days. For image analysis of the droplets, images of the spheroids within the droplets were taken using a microscope (IX73, Olympus, Japan) and analyzed using ImageJ software (National Institute of Health, USA). To quantify the spheroid morphology, 151 droplets were measured, and ShI was calculated according to the following formula:

$${\rm{ShI}} = \frac{{4\pi A}}{{P^2}}$$

where *A* and *P* are the area and perimeter of the cells, respectively.

### Synthesis and characterization of the rGO-BPEI-PEG nanocomposites

GO solution was obtained from Graphene Square, Inc., Korea. BPEI (MW 2 kDa) was purchased from Tokyo Chemical Industry Co. Ltd. (Tokyo, Japan). Carboxylated PEG (MW 5000 Da, PEG-COOH) was provided by Nanocs, Inc. (New York, USA). 1-Ethyl-3-(3-dimethylaminopropyl) carbodiimide (EDC), N-hydroxysuccinimide (NHS), sodium hydroxide, and chloroacetic acid were purchased from Sigma-Aldrich (USA). Carboxylated GO (GO-COOH) was synthesized using a modified method according to a previous report^21^. Briefly, to convert the excess hydroxyl groups on the GO nanosheets into carboxyl groups, 20 mL of GO solution (1 mg/mL) was ultrasonicated for 1 h. Furthermore, NaOH (2.4 g) and chloroacetic acid (2 g) were dissolved in the GO solution. The mixture was treated by additional ultrasonication for 3 h. GO-COOH was dialyzed against a dialysis membrane (MWCO 3,500 Da, Sigma-Aldrich, USA) in deionized water for 2 days to remove dispensable ions. Finally, GO-COOH was obtained by freeze-drying for 46 h. BPEI was covalently conjugated to the carboxyl group of GO using EDC/NHS chemistry^26,35^. EDC (151 mg, 1 mmol) and NHS (115 mg, 1 mmol) were added to a GO-COOH solution (15 mg/mL) in a vial, and TEA (200 µL) was added to a BPEI solution (300 mg) in deionized water. Next, the BPEI solution was added to the GO-COOH solution, and the mixture was sonicated for 3 h and then stirred for 24 h at room temperature. The resulting GO-BPEI was dialyzed by a dialysis membrane (MWCO 3500 Da) in deionized water for 2 days to remove the unreacted BPEI, TEA, and coupling reagents. To enhance the colloidal stability of GO-BPEI, PEG-COOH was conjugated to the end of the primary amine of BPEI with the same reaction. PEG-COOH was dissolved in 10 mL of phosphate-buffered saline (PBS) (pH 5.8) containing EDC (120 mg) and NHS (135 mg), and the mixture was sonicated for 30 min. GO-BPEI solution (5 mg/mL) was slowly added to the activated PEG solution. After reaction for 18 h at room temperature with stirring, the PEG-conjugated GO-PEI (GO-BPEI-PEG) was dialyzed by a dialysis membrane (MWCO 6-8 kDa) against deionized water. The reducing process of GO-BPEI-PEG was conducted by treatment with 0.05 v/v% hydrazine monohydrate (80%) followed by heating to 80 °C for 15 min. rGO-BPEI-PEG was obtained by freeze-drying. Atomic force microscopy (AFM, XE-100, Park Systems, Korea) was employed to characterize the morphology and size of the rGO-BPEI-PEG nanocomposites. The chemical bonding of GO-BPEI-PEG and conjugation of BPEI-PEG to reduced GO was confirmed by Fourier transform infrared spectroscopy (FT-IR, Nicolet 6700, Japan) using KBr pellets at room temperature in the range of 4000–400 cm^−1^ at a resolution of 4 cm^−1^. The photothermal properties of rGO-BPEI-PEG were confirmed by measuring the absorbance at 800 nm using UV-visible spectroscopy (UV 1800, Shimadzu, Japan). The surface charges of GO-COOH, GO-BPEI, GO-BPEI-PEG, and rGO-BPEI-PEG were determined by zeta potential measurements using a Zetasizer Nano Z (Malvern Instruments, UK).

### Cell viability and cellular uptake analysis

To confirm cell viability, spheroids were incubated with calcein AM (2 μM Sigma, USA) and ethidium homodimer-1 (EthD-1, 4 μM, Life Technologies, USA) at 37 °C for 15 min after being replated. The photothermal effects of the rGO-BPEI-PEG nanocomposites were tested by irradiating a solution of the nanocomposite (60 μg/mL) using a NIR laser (808 nm, 1 W/cm^2^, BWF2, B&W, Denmark) while monitoring the temperature of the solution. Next, cellular uptake of the nanocomposite was tested. For this, the spheroids generated by the microfluidic device were replated onto 8-well plates (ibidi, Germany), and single tumor cells (1×104 cells/mL) were seeded onto an 8-well plate and cultured for 24 h. After 24 h, 60 μg/mL rGO-BPEI-PEG nanocomposite was added to the medium. Additionally, after 4 h of further incubation, spheroids were fixed using 4% paraformaldehyde (Sigma-Aldrich, USA) after a washing step. Alexa Fluor 594 phalloidin (Thermo Fisher Scientific, USA) and 4’,6-diamidino-2-phenylindole (DAPI, Thermo Fisher Scientific, USA) were used to stain the spheroids according to the manufacturer’s instructions. Cellular uptake was shown using confocal fluorescence microscopy images (LSM 710, Carl Zeiss, Germany).

### PTT effects of the rGO-BPEI-PEG nanocomposites

To demonstrate the photothermal effects on the spheroids after uptake of the nanocomposites, tumor spheroids were replated onto a cell culture plate and incubated for one additional day. The culture medium was exchanged with medium containing 1 v/v% rGO-BPEI-PEG nanocomposites, and the spheroids were incubated for an additional 4 h. Then, the spheroids were irradiated with a NIR laser (1 W, 808 nm) for 10 min. The cell viability of the U87MG brain tumor spheroids was evaluated using a live/dead assay kit (Thermo Fisher, USA) according to the manufacturer’s instructions. Live/dead staining results were evaluated using a fluorescence microscope (IX73, Olympus, Japan). To perform FACS analysis of the tumor spheroids, the tumor spheroids were harvested from the culture plate and subsequently dissociated into single cells using 0.25% trypsin-EDTA for 3 min after photothermal therapy. Afterward, the dyes (e.g., calcein AM and ethidium homodimer-1) were used to stain the live and dead cells before FACS analysis. FACS analysis was conducted with an LSR II analyzer (BD Biosciences, USA). FACS results were visualized using Flow-Jo software (BD Bioscience, USA).

### Drug screening of uniform-sized neurospheres

NSC-derived neurospheres were differentiated for 7 days and treated with 1 μM thapsigargin neurotoxin for 6 h. Afterward, thapsigargin was rinsed with DPBS. The neurospheres were fixed with 4% paraformaldehyde for 15 min. They were subsequently treated with 0.1% Triton X-100 (Samchun, Korea) for 5 min and 6% bovine serum albumin (BSA, Sigma-Aldrich, USA) for 2 h at room temperature. Next, the neurospheres were treated with the neuronal-specific class III beta-tubulin (1:1000, Tuj1, Stem Cell Technology, Canada) primary antibody at 4 °C overnight. Next, the neurospheres were rinsed with DPBS twice and incubated with Alexa Fluor® 488 secondary antibodies (Invitrogen, USA) for 4 h at room temperature. After two washes in DPBS, DAPI was applied for nuclear staining. Quantitative analysis of the neurite networks was performed using ImageJ software.

## Supplementary information

Supplementary Material

Supplementary Video 1
