# Supplementary material for: Generation of tumor spheroids using a droplet-based microfluidic device for photothermal therapy
Source: Microsyst Nanoeng. 2020 Jun 29;6:52. doi: 10.1038/s41378-020-0167-x (PMC8433304; doi:10.1038/s41378-020-0167-x)
Supplement: Supplementary file 1 — Supplementary Material [file 41378_2020_167_MOESM1_ESM.docx]

**Supplementary Material**


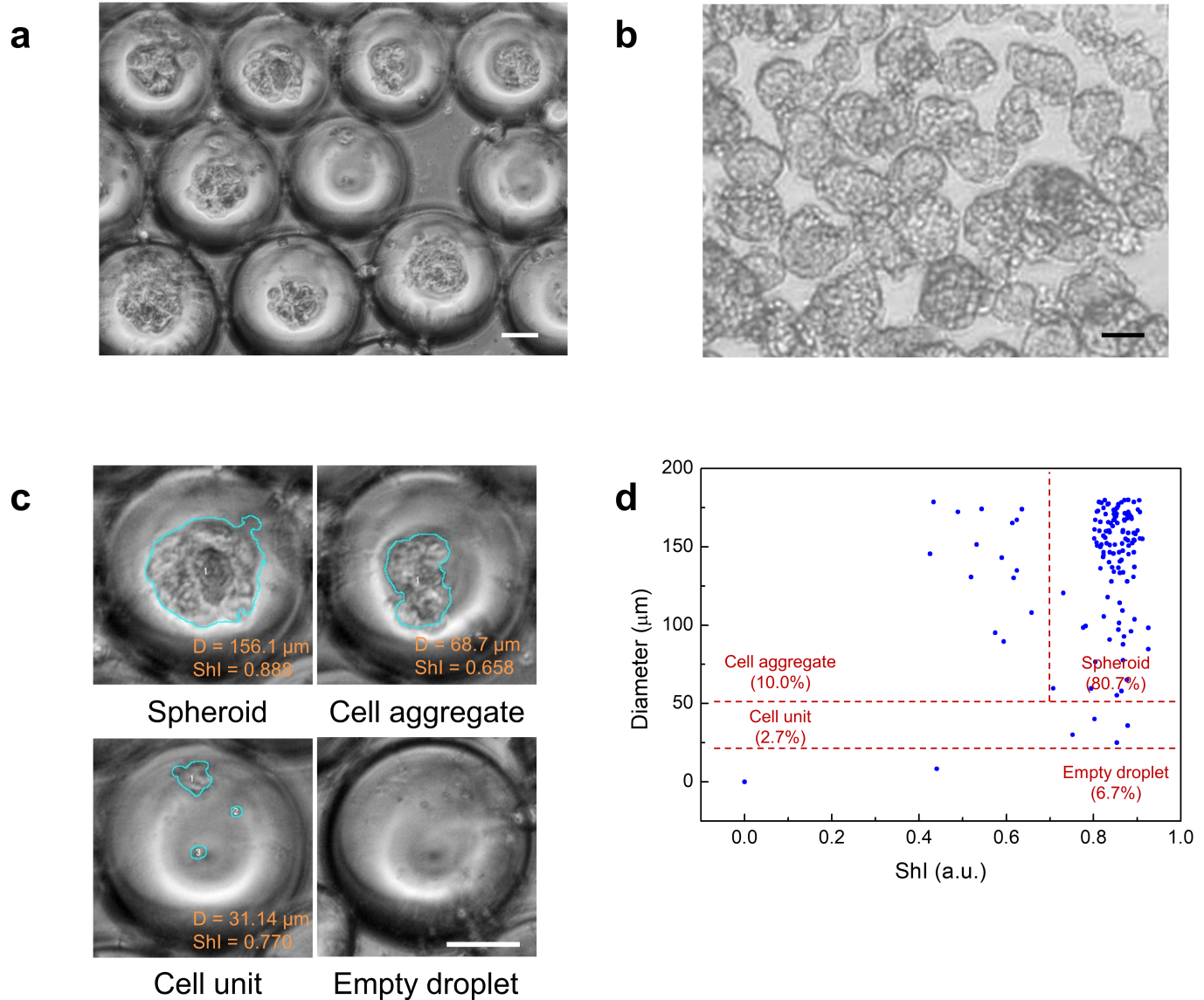


**Fig. S1** Morphological analysis of tumor spheroids. **a** Microscope image of the tumor spheroids generated from droplet-based microfluidic device. **b** Microscope image of tumor spheroids after re-plate. Scale bars are 100 µm. **c** Representative images for a spheroid, cell aggregate, cell unit, and empty droplet. The blue line indicates the edge of the detected cells. **d** Plot analysis of all cells (n=151) with circularity (*Shl*) and diameter. Dashed-lines show the median of the corresponding data.


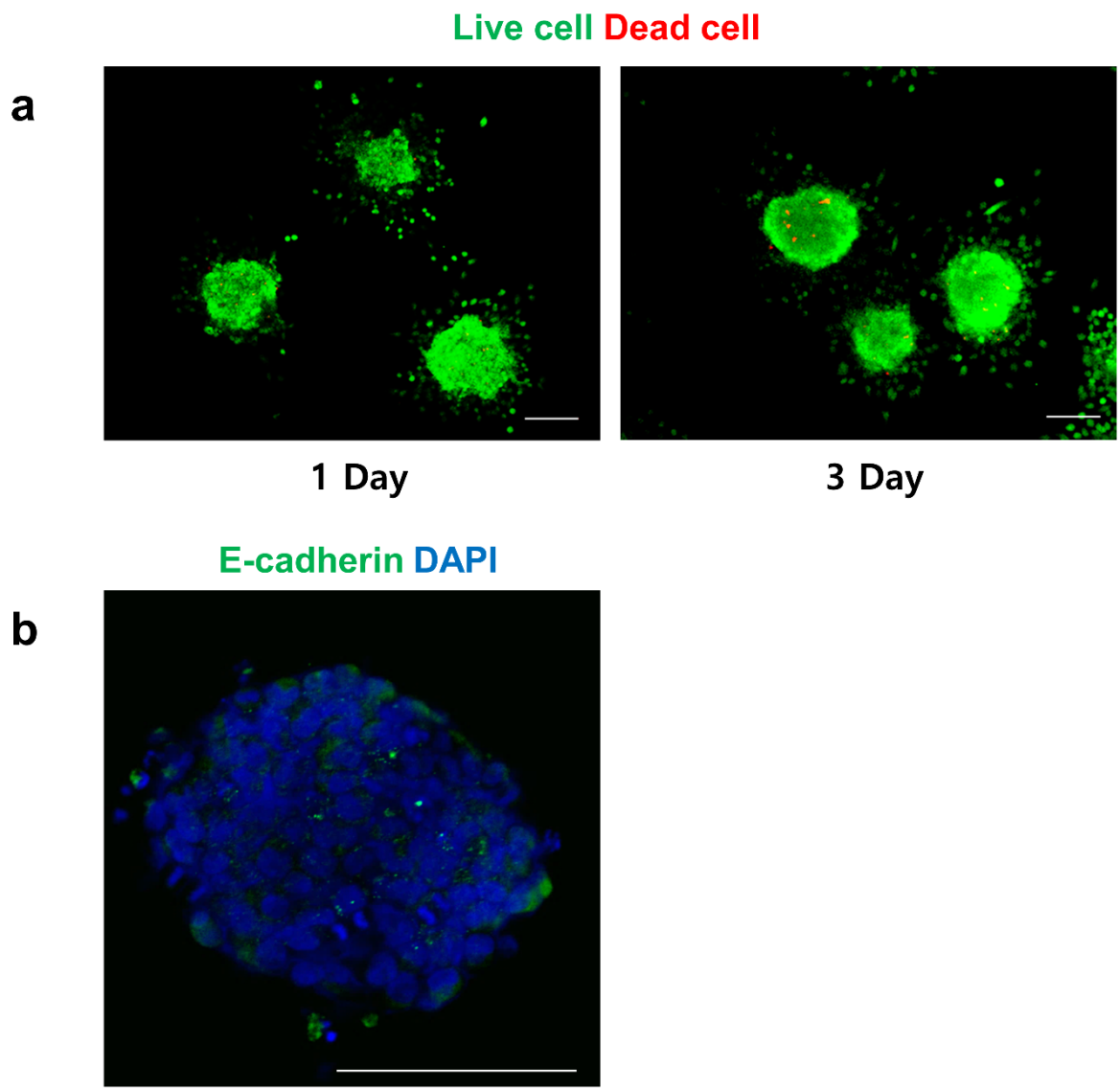


**Fig. S2** Fluorescent images of tumor spheroids. **a** Viability of tumor spheroids after re-plate. The live and dead cells are stained with calcein AM (green) and ethidium homodimer (red) on day 1 and day 3 after re-plate. **b** Confocal laser scanning microscopy image of brain tumor spheroids. Spheroids are immunostained by E-cadherin (green) and cell nuclei are stained by DAPI (blue). Scale bars are 100 µm.


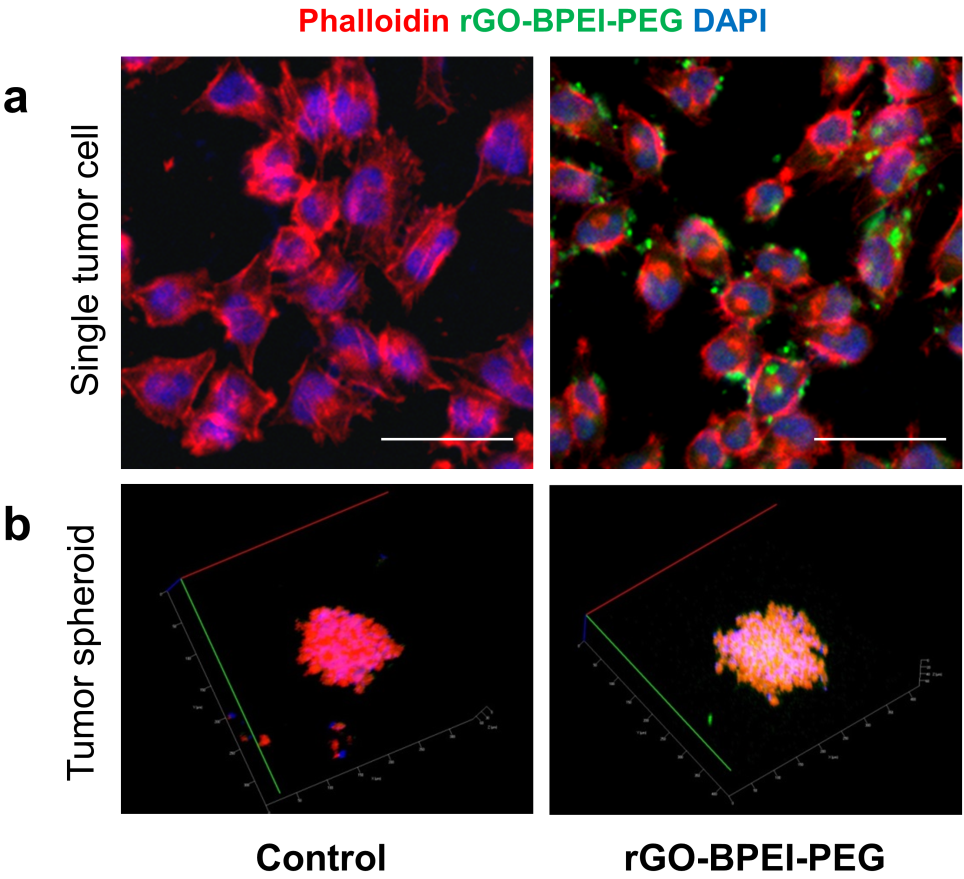


**Fig. S3** Confocal laser scanning microscopy image of cellular uptake. **a** Confocal laser scanning microscopy image of single tumor cells treated with rGO-BPEI-PEG nanocomposites. **b** Z-stack confocal laser scanning microscopy image of 3D tumor spheroids treated with rGO-BPEI-PEG nanocomposites. Scale bars are 100 μm.


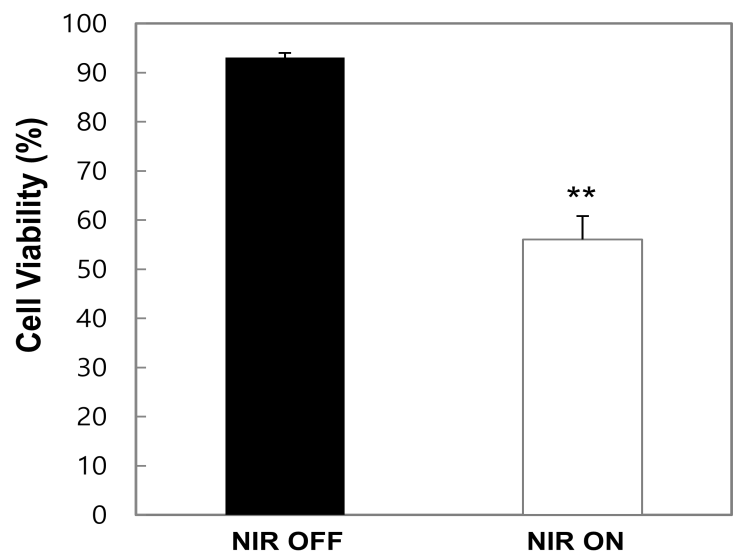


**Fig. S4**. Viability analysis of tumor spheroids in response to NIR laser irradiation.

**Supplementary Video 1.** Video showing droplet generation yield at an oil flowrate of 10-50 µL/min and a water flowrate of 5 µL/min. For the video, the continuous phase consists of fluorinated oil with 2% diluted surfactant. For dispersed phase DI water with 10 v/v% blue food dye is used. Generation yield of the slowest condition (10 µL/min) is 14 Hz and the fastest condition (50 µL/min) is 70 Hz.

| **3D spheroid generation method**  **[ref]** | **Advantages** | **Limitations** | **Through-put** | **Spheroid size control** | **Automation** |
| --- | --- | --- | --- | --- | --- |
| Non-adherent surface  [1-3] | - Easy accessibility  - Long-term culture | - Low-  throughput  - Variations in spheroid size | 96-384 spheroids  in one time | x | X |
| Hanging drop technique  [4-7] | - High reproducibility  - Easy operation | Incompetency for exchanging cell medium | 96-384 spheroids in one time | - Sample  volume  - Cell  concentration | △ |
| Micro-structure  [8-9] | - Uniform size  - Long-term culture | Difficult control of spheroid size | 25-1,024 spheroids  in one time | - Size of  structure  - Culture day  - Cell  concentration | X |
| Droplet-based microfluidic system | - Controllable size  - Rapid generation  - Short handling time | Complex spheroid collection | 42,000 spheroids  within 10 minutes | - Flowrates of  injected  solution  - Cell  concentration | O |

**Supplementary Table S1.** Comparison table of 3D spheroid generation methods.

**References**

1 Svirshchevskaya, E. *et al.* Characteristics of multicellular tumor spheroids formed by pancreatic cells expressing different adhesion molecules. *Life Sci.* **219**, 343-352 (2019).

2 Rustamov, V., Keller, F., Klicks, J., Hafner, M. & Rudolf, R. Bone sialoprotein shows enhanced expression in early, high-proliferation stages of three-dimensional spheroid cell cultures of breast cancer cell line MDA-MB-231. *Front. Oncol.* **9**, 36 (2019).

3 Tsai, M.-T., Huang, B.-H., Yeh, C.-C., Lei, K. F. & Tsang, N.-M. Non-Invasive Quantification of the Growth of Cancer Cell Colonies by a Portable Optical Coherence Tomography. *Micromachines* **10**, 35 (2019).

4 Han, Q. *et al.* Tumor cell‑fibroblast heterotypic aggregates in malignant ascites of patients with ovarian cancer. *Int. J. Mol. Med.* **44**, 2245-2255 (2019).

5 Zhao, L. *et al.* A 3D Printed Hanging Drop Dripper for Tumor Spheroids Analysis Without Recovery. *Sci. Rep.* **9**, 1-14 (2019).

6 Bartosh, T. J. & Ylostalo, J. H. Preparation of anti‐inflammatory mesenchymal stem/precursor cells (MSCs) through sphere formation using hanging‐drop culture technique. *Curr. Protoc. Stem Cell Biol.* **28**, 2B. 6.1-2B. 6.23 (2014).

7 Aijian, A. P. & Garrell, R. L. Digital microfluidics for automated hanging drop cell spheroid culture. *J. Lab. Autom.* **20**, 283-295 (2015).

8 Chen, Y.-C., Lou, X., Zhang, Z., Ingram, P. & Yoon, E. High-throughput cancer cell sphere formation for characterizing the efficacy of photo dynamic therapy in 3D cell cultures. *Sci. Rep.* **5**, 12175 (2015).

9 Choi, J. W., Lee, S.-Y. & Lee, D. W. A Cancer Spheroid Array Chip for Selecting Effective Drug. *Micromachines* **10**, 688 (2019).
